# Dynamic changes in *Ccn3* expression across the limbic forebrain through the mouse estrous cycle and during lactation

**DOI:** 10.1111/jne.70050

**Published:** 2025-05-27

**Authors:** Shel‐Hwa Yeo, Zulfiye Gul, Ziyue Zhou, Leila Muresan, Ellen G. Wall, Allan E. Herbison

**Affiliations:** ^1^ Department of Physiology, Development and Neuroscience University of Cambridge Cambridge UK; ^2^ Department of Pharmacology, School of Medicine Dokuz Eylul University Izmir Turkey; ^3^ Cambridge Advanced Imaging Centre University of Cambridge Cambridge UK; ^4^ School of Computing and Information Science Anglia Ruskin University Cambridge UK

**Keywords:** amygdala, *ccn3*, estrous cycle, hypothalamus, kisspeptin, lactation

## Abstract

Cellular communication network factor 3 (CCN3), also known as nephroblastoma overexpressed (NOV), is an adipocytokine that has recently been suggested to be secreted selectively by hypothalamic arcuate nucleus kisspeptin (ARN^KISS^) neurons to protect bone density during lactation. Using RNAscope hybridization, we have examined the expression of *Ccn3* transcripts in the forebrain of male mice and female mice across the estrous cycle and during lactation. Transcripts for *Ccn3* are highly expressed in the cerebral cortex, hippocampus, subthalamic nucleus, and amygdala in both sexes. Lower levels of *Ccn3* mRNA were detected within the hypothalamus of females but not males. During lactation (day 11), a substantial 6‐fold increase in the numbers of cells expressing *Ccn3* mRNA was found in the arcuate and dorsomedial nuclei of the hypothalamus as well as the posterodorsal division of the medial amygdala. Approximately 50% of cells expressing *Ccn3* in the ARN during lactation also contained *Kiss1* transcripts. An increase in *Ccn3* mRNA expression in ARN^KISS^ neurons also occurred during proestrus. These observations demonstrate that multiple limbic brain regions and cell types coordinately up‐regulate their expression of *Ccn3* during lactation in the mouse.

## 1 INTRODUCTION

Cellular communication network factor 3 (CCN3) is part of a family of six secreted matricellular peptides involved in the local regulation of extracellular signaling.^1^, ^2^ Initially identified as being over‐expressed in nephroblastomas and named nephroblastoma overexpressed (NOV),^3^ it is now apparent that CCN3 is widely expressed throughout the body including adipocytes, myocytes, glandular epithelia, and immune cells.^1^ Although relatively little is known about CCN3 within the CCN family, it has been proposed to have a range of anti‐proliferative effects on chondrocytes, osteoblasts, vascular smooth muscle cells, and tumors, while promoting angiogenesis and hematopoiesis.^1^ It is particularly interesting that CCN3 can be measured in the circulation, with high levels having strong associations with obesity, diabetes, and inflammation.^4^, ^5^, ^6^ The source of plasma CCN3 is thought to be from adipose tissue, and CCN3 is often referred to as an adipocytokine.^2^, ^7^, ^8^, ^9^

It was recently proposed that CCN3 may also operate as a neuroendocrine hormone that is released from hypothalamic arcuate nucleus kisspeptin (ARN^KISS^) neurons at the time of lactation to maintain bone density.^10^ Prior studies had shown that the deletion of estrogen receptor alpha (ESR1) from forebrain neurons with either Nestin‐cre or Nkx2.1‐cre drivers resulted in mice with a robust increase in bone density.^11^, ^12^ Importantly, Babey and colleagues found that the knockdown of *Ccn3* expression within the ARN of female *Esr1* ^*Nkx2.1‐cre*^ mice protected against this increase in bone density.^10^ Kisspeptin neurons were considered to be the likely neuronal phenotype within the ARN as they were reported to selectively express CCN3 during lactation^10^ and the embryonic deletion of ESR1 from all *Kiss1* cells results in an increased bone mass phenotype,^11^ alongside precocious puberty.^13^

The ARN^KISS^ neurons operate as the GnRH pulse generator^14^, ^15^ but also send projections to multiple limbic brain regions where their functions remain unknown.^16^, ^17^ The proposal that ARN^KISS^ neurons may have an additional neuroendocrine role through the expression of CCN3 specifically during lactation is intriguing. However, as studies to date have only used *Esr1* ^*Nkx2.1‐cre*^ mice to explore CCN3 function,^10^ there is as yet no direct evidence that CCN3 derived from ARN^KISS^ neurons is involved in bone remodeling. In particular, the selectivity of *Ccn3* expression to kisspeptin neurons within the ARN or to lactation remains unclear. To begin to resolve some of these issues, we have examined the expression of *Ccn3* transcripts within the ARN and other limbic brain regions in male mice and in females across the estrous cycle and during lactation.

## 2 MATERIALS AND METHODS

### 2.1 Animals

C57BL/6J male and female mice (>8 weeks old; Charles River, UK) were group housed in individually ventilated cages under controlled conditions (12:12 h light/dark cycle, lights on at 05:30 h; 22 ± 2C; humidity 45–55%) with environmental enrichment and ad libitum access to food (RM1‐P, SDS, UK) and water. All experimental protocols were approved by the University of Cambridge Animal Welfare and Ethics Review Body under the UK Home Office license P174441DE. Estrous cycles were determined by daily vaginal smears at approximately 10 am for at least three consecutive estrous cycles and culled at 10 am on the required day of the estrous cycle. The reproductive hormone profiles of the cycling female mice (*N* = 4–5/estrous stage) used in this study have been reported.^18^ Lactating mice were culled on day 11 of lactation between 10 am and noon.

### 2.2 Brain RNAScope®

A lethal dose of pentobarbital (Dolethal, 400 mg/kg, i.p; Vetoquinol UK) was administered and blood was collected for hormone analysis as reported^18^ and the brains were removed and immersed in 4% paraformaldehyde in 0.1 M phosphate‐buffered saline overnight before being placed in 30% sucrose. Brains were then embedded in optimal cutting temperature compound and sets of 16 μm‐thick coronal sections containing the medial basal hypothalamus were cut on a cryostat (Bright Instruments, UK) and mounted on glass slides and stored at −80 C.

Multiplexed fluorescent in situ hybridization using RNAScope® (ACD, Multiplex Fluorescent V2) was performed according to the manufacturer's protocol using the probes for Ccn3/Nov (ACD, #415341‐C2) and Kiss1 (ACD, #500141‐C1). In brief, sections on glass slides were baked at 60°C for 30 min before being fixed in 50%, 70%, 100% ethanol for 5 min. Slides underwent hydrogen peroxidase blocking for 10 min at room temperature and then were immersed in target retrieval reagent for 15 min at 95–100°C. Slides were then immersed immediately in water and pretreated with RNAscope Protease III for 25 min at 40°C. Sections were then incubated in a hybridization probe for 2 h at 40°C. Following three amplification steps, hybridized probes were reacted with TSA plus Opal 520 and (AKOYA, Cat. No. FP1487001KT) Opal 570 system (AKOYA, Cat. No. FP1488001KT) for 30 min at 40°C and then stained with Hoescht dye 33,342 in 1:2000 (H1399, Invitrogen, US) for 10 min at room temperature and cover‐slipped with VECTASHIELD Vibrance Antifade mounting medium (Vector, H‐1000).

### 2.3 Confocal imaging

All images were acquired with a laser scanning confocal microscope (Zeiss LSM 710) using appropriate excitation and emission filters, a pinhole of 1 AU, and a 40× oil immersion objective. As the ARN, dorsomedial nucleus (DMN), and posterodorsal medial amygdala (MEPD) all exhibited obvious increases in *Ccn3* mRNA expression at lactation, they were selected for quantitative analysis. Three sections were selected for each brain region in each animal by an investigator blind to their identity, with analysis undertaken bilaterally. Images were taken with a 0.75× zoom (0.553 μm/pixel), 512 × 512 pixels, and in z‐stacks with 2 μm steps.

### 2.4 Analysis pipeline

**FIGURE 1:**
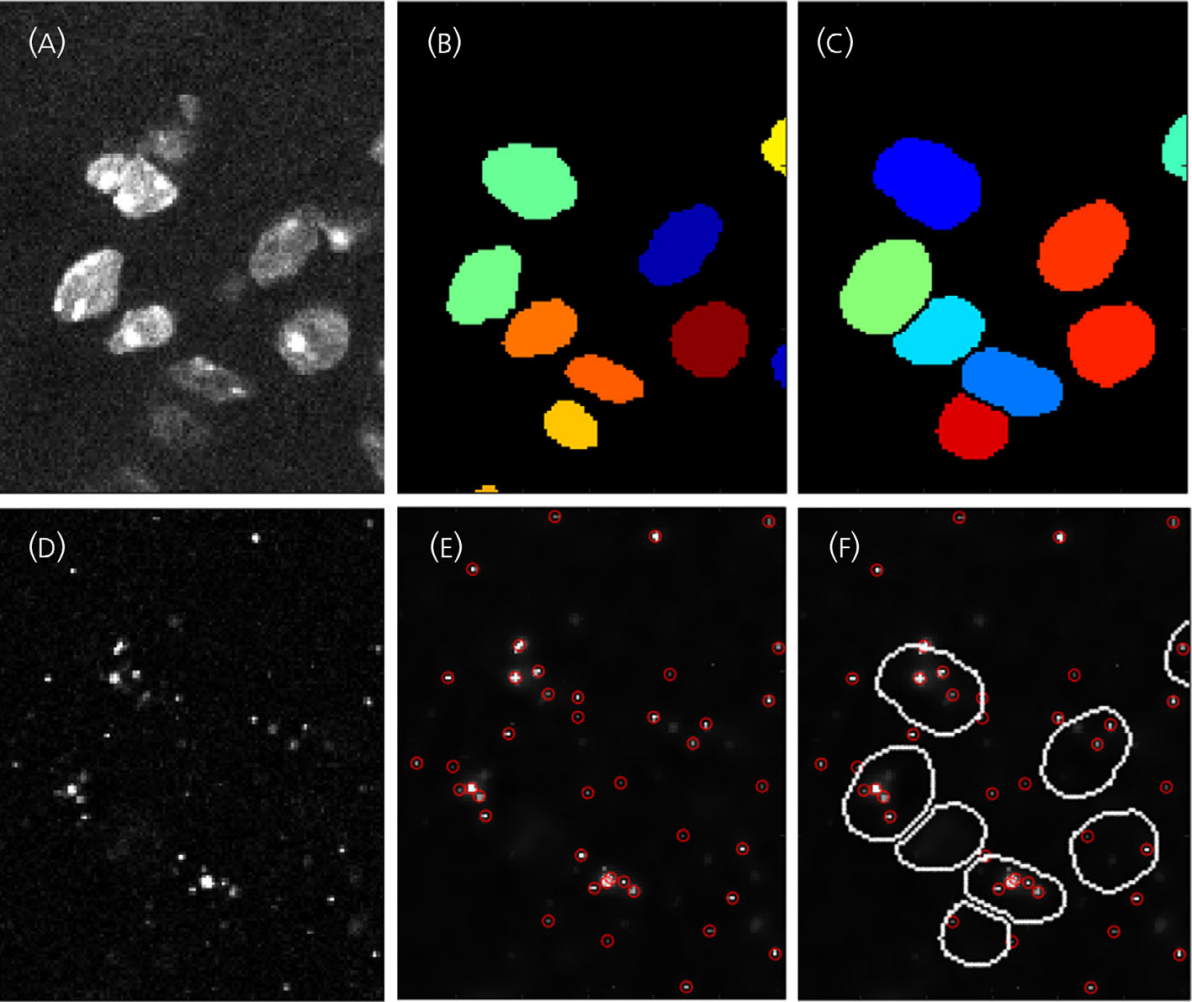
RNAscope image analysis pipeline. The nuclei of Hoescht‐stained cells are visualized (A) and labeled using Stardist segmentation (B). Each region of interest is dilated by 2 pixels (C) to capture the associated mRNA molecules in the cell cytoplasm. RNAscope signal is imaged across the same section (D) and detected as “dots” (E) and then quantified per each region of interest (F).

Confocal images were processed with a new RNAscope signal analysis pipeline (https://github.com/lemur01/CountRNAScope) based on a MATLAB (The MathWorks Inc., USA) script combined with a deep machine learning algorithm, StarDist^19^ that is called through an ImageJ/FIJI plugin (National Institutes of Health, USA), TrackMate^20^ to identify cell nuclei‐delineated regions of interest (ROI) in 3D based on nuclei segmentation of the Hoescht signal (third channel) (Figure 1A–F). The main steps of the workflow consist of first, delineating the cell nuclei ROIs and second, counting the mRNA molecules (RNAscope “dots”) related to the delineated nuclear regions. Each cell nuclei ROI is dilated by two pixels from the nucleus (~0.83 μm) to capture the associated cytoplasmic mRNA molecules (Figure 1C). The RNAscope dots inside the single cell‐delineated ROIs are detected and counted in channels one and two (Figure 1F). The RNAscope dots were enhanced and candidates detected using an *à trous* wavelet transform as described previously^21^, ^22^ with a threshold of 10 analog digital units.

After processing the complete set of confocal brain images through the analysis pipeline, the distribution of RNAscope dot expression/ROI was established for each probe and the threshold for determining a positive cell set at the mean minus two standard deviations. This resulted in threshold values of 10 dots for *Kiss1* and 7 dots for *Ccn3‐expressing* cells. All cells expressing dots above these thresholds were counted as positive cells. Confocal images from the experiment were processed in a single pipeline run.

### 2.5 Statistical analysis

Statistical analysis was carried out using Prism 10 (GraphPad software Inc., USA). Given that a normal distribution could not be demonstrated with the N numbers employed, non‐parametric Kruskal–Wallis ANOVA was used, followed by Dunn's multiple comparisons tests. *p* < .05 was considered significant.

## 3 RESULTS

### 3.1 Regional Ccn3 mRNA expression in diestrous female and male mice

**FIGURE 2:**
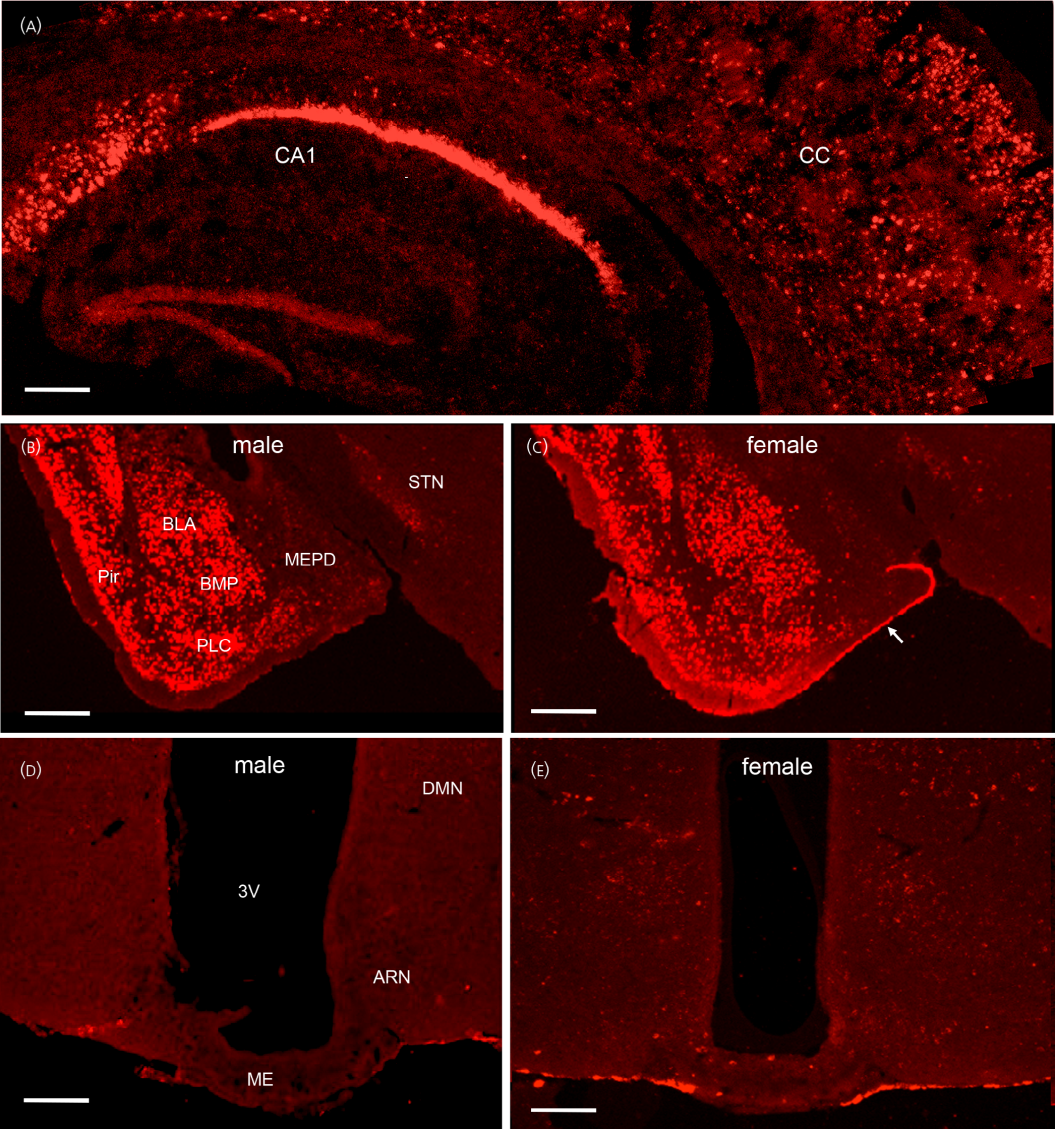
Expression of *Ccn3* transcripts in the male and female mouse brain. Low‐power views of the cerebral cortex (CC) and CA1 hippocampus in a diestrous female mouse (A), the amygdala and surrounding regions in male (B) and diestrous female (C) mice and the mediobasal hypothalamus in a male (D) and diestrous female mouse (E). C, arrow indicates *Ccn3* mRNA expression in the meningeal layer when present on sections. 3V, third ventricle; ARN, arcuate nucleus; BLA, basolateral amygdala; BMP, basomedial amygdala; DMN, dorsomedial nucleus; ME, median eminence; MEPD, posterodorsal medial amygdala; Pir, pyriform cortex; STN, subthalamic nucleus. Scale bars represent 250 μm (A), 400 μm (B, C), and 250 μm (D, E).

Cells expressing high levels of *Ccn3* mRNA were detected throughout the cerebral cortex (Figure 2A), CA1 hippocampus (Figure 2A), subthalamic nucleus (Figure 2B,C) and widely within the amygdala in both males (*N* = 4) and diestrous‐stage females (*N* = 4) (Figure 2B,C). Expression was especially robust in the basolateral and basomedial divisions of the amygdala and the adjacent piriform cortex of both sexes (Figure 2B,C). In female mice, smaller numbers of scattered cells expressing *Ccn3* mRNA were detected within the mediobasal hypothalamus, including the ARN and DMN (Figure 2E) as well as in the posterior hypothalamus. In contrast, no detectable *Ccn3* mRNA‐expressing cells were observed in the mediobasal or posterior hypothalamus of the male mice (Figure 2D). There was also significant *Ccn3* mRNA expression in the meningeal lining of the brain, when present (Figure 2C). Positive control RNAscope probes for *Ppib* and *Polr2a* always provided robust signals, whereas the negative control probe against the bacterial gene *DapB* never resulted in a positive hybridization signal.

**FIGURE 3:**
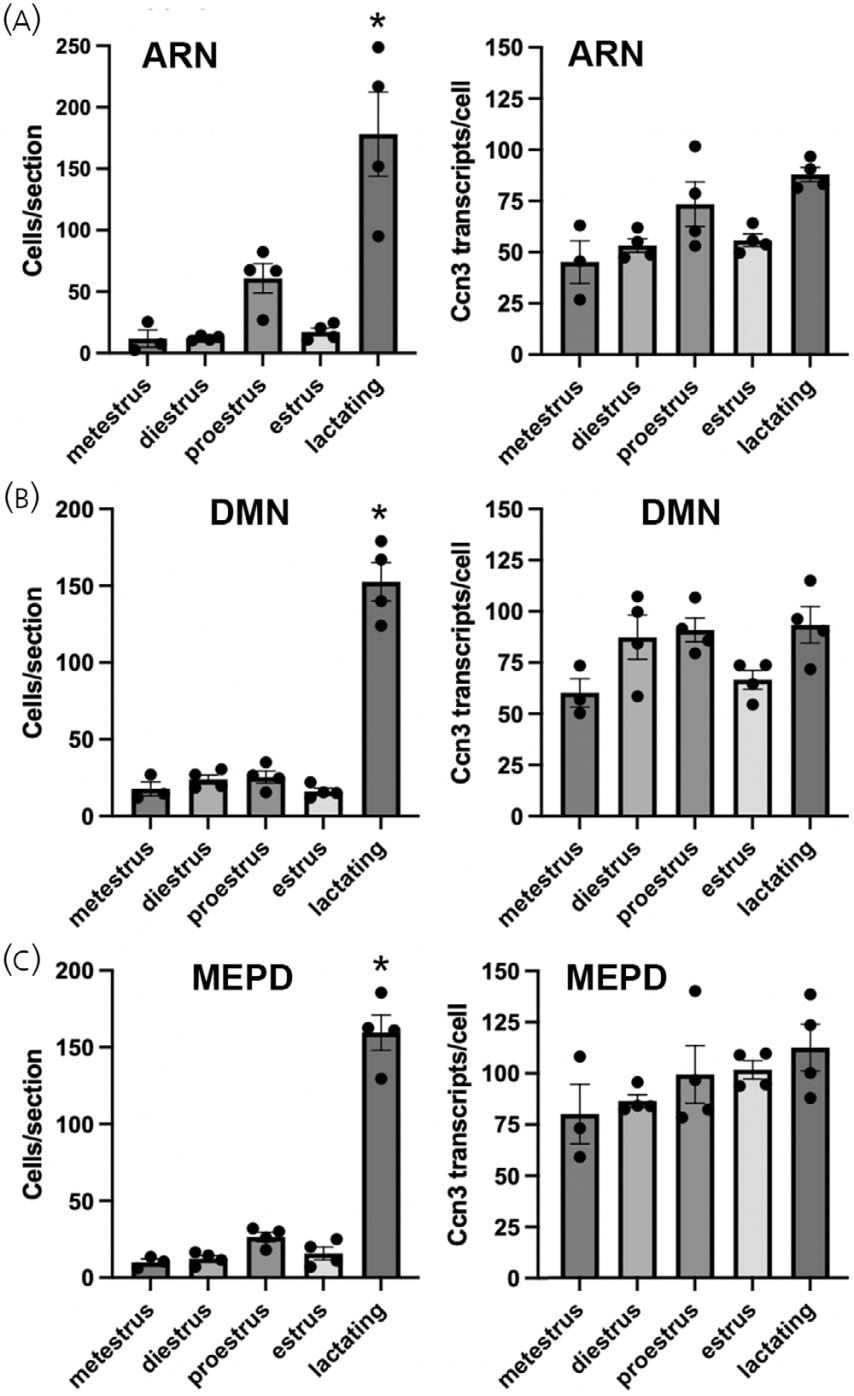
Expression of *Ccn3* mRNA across the estrous cycle and at lactation (day 11). Histograms showing the mean ± SEM numbers of cells expressing *Ccn3* transcripts (left) and the mean ± SEM expression per cell (right). (A) Arcuate nucleus (ARN), *p < .05 compared to metestrus and diestrus. (B) Dorsomedial nucleus (DMN), *p < .05 compared to metestrus and estrus. (C) posterodorsal division of the medial amygdala (MEPD), *p < .05 compared to metestrus and diestrus. *N* = 4 in all cases except metestrus (*N* = 3). Kruskal–Wallis with post‐hoc Dunn's tests.

Based on observations in lactating mice (below), we undertook a quantitative analysis of *Ccn3* mRNA expression in the ARN, DMN, and MEPD of female mice across the estrous cycle. This revealed no significant differences in the numbers of *Ccn3* mRNA‐expressing cells or *Ccn3* mRNA/cell in the ARN, DMN, or MEPD (Figure 3A–C). There was, however, a non‐significant trend for increased numbers of *Ccn3* mRNA‐expressing cells in the ARN on proestrus (*N* = 4) compared to metestrus (*N* = 3) and diestrus (*N* = 4) (Kruskal–Wallis 9.049, *p* = .0101, Dunn's *p* = .0575 (metestrus), *p* = .0676 (diestrus); Figure 3A).

**FIGURE 4:**
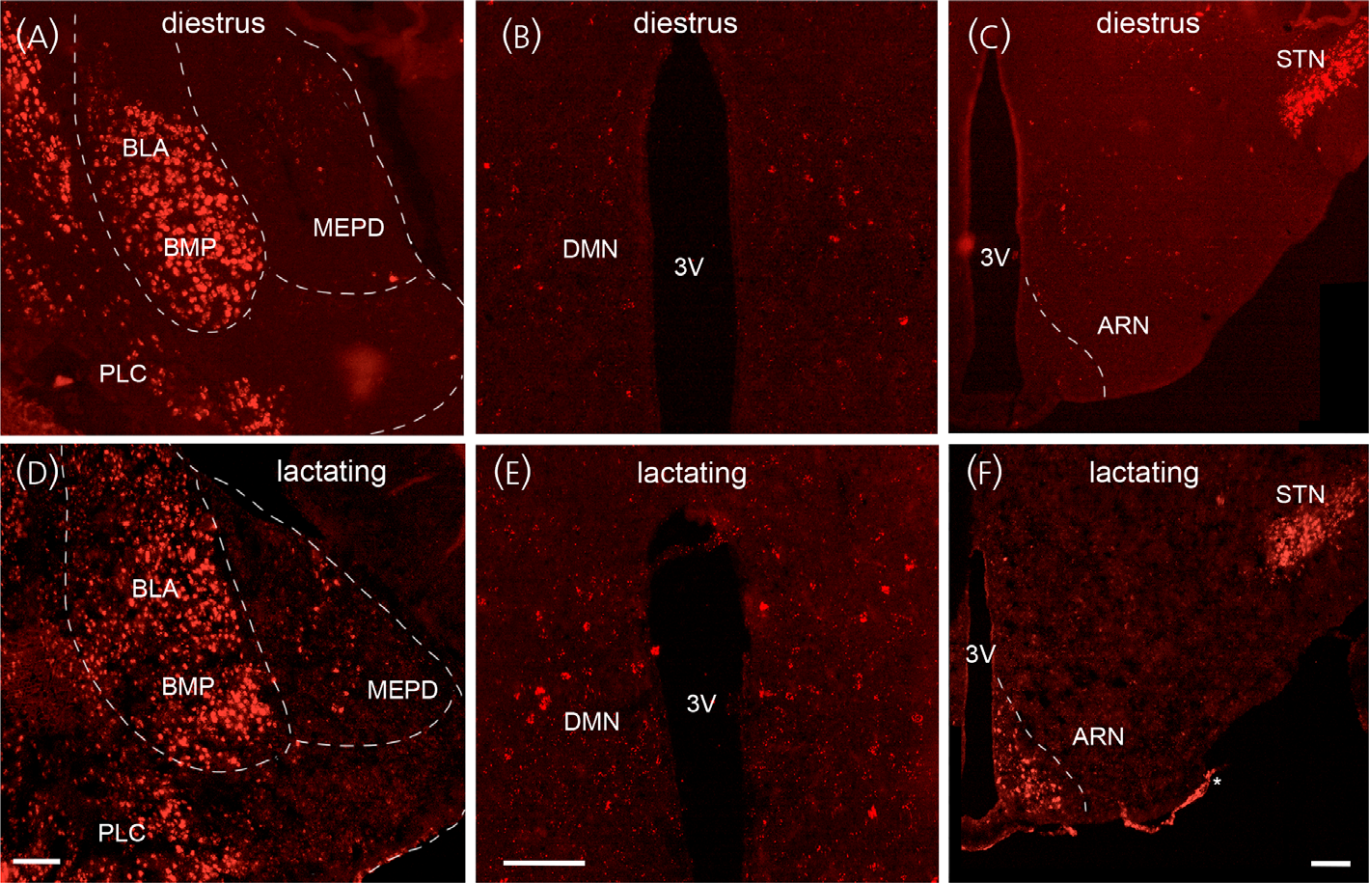
Expression of *Ccn3* transcripts in the female mouse brain in diestrus and lactation. Low‐power views of *Ccn3* mRNA expression in the amygdala and mediobasal hypothalamus of diestrous (A–C) and lactating (D–F) mice. In the amygdala, consistent high expression is found in the basolateral (BLA) and basomedial amygdala (BMA), and posterior lateral cortical amygdala (PLC) while *Ccn3* transcripts are increased in the posterodorsal medial amygdala (MEPD) of lactating mice. An increase in *Ccn3* mRNA expression occurs in the dorsomedial (DMN) (B, E) and arcuate nuclei (ARN) (C, F) of lactating mice. 3V, third ventricle, STN, subthalamic nucleus. Scale bars represent 200 μm.

In day 11 lactating mice, the expression of *Ccn3* mRNA was significantly elevated in the ARN, DMN and MEPD (Figures 3A–C and 4A–F). In all regions, there was a marked increase in the numbers of *Ccn3* mRNA‐expressing cells while *Ccn3* transcript levels per cell remained statistically unchanged (Figure 3A–C, *N* = 4 all groups). In the ARN, the numbers of *Ccn3*‐positive cells increased from 12.3 ± 1.0 cells/section in diestrus to 178.2 ± 34.3 in lactating mice (Kruskal–Wallis 14.72, *p* = .0053, Dunn's *p* = .0245 (diestrus), *p* = .0280 (metestrus), Figure 3A). In the DMN, the numbers of *Ccn3*‐positive cells increased from 16.3 ± 2.1 cells/section in estrus compared to 152.5 ± 12.5 in lactating mice (Kruskal–Wallis 12.11, *p* = .0166, Dunn's *p* = .0166 (estrus), Figure 3B). In the MEPD, the numbers of *Ccn3*‐positive cells increased from 12.8 ± 2.1 cells/section in diestrus compared to 159.6 ± 11.5 in lactating mice (Kruskal–Wallis 14.04, *p* = .0072, Dunn's *p* = .0206 (diestrus), *p* = .0067 (metestrus), Figure 3C).

### 3.2 Ccn3 mRNA expression in ARN Kiss1 neurons in female mice

**FIGURE 5:**
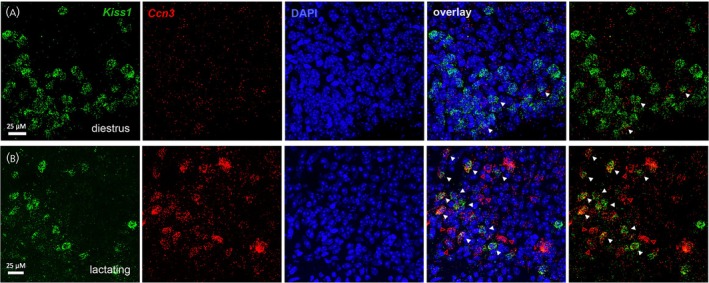
Multiplex RNAscope showing *Kiss1* (green) and *Ccn3* (red) expression in the ARN of a diestrous female mouse (A) and day 11 lactating dam (B). White triangles in the “overlay” indicate Kiss1 neurons expressing *Ccn3* transcripts visible in the plane of the section while the open red triangles show examples of non‐*Kiss1* cells expressing *Ccn3* mRNA.

**FIGURE 6:**
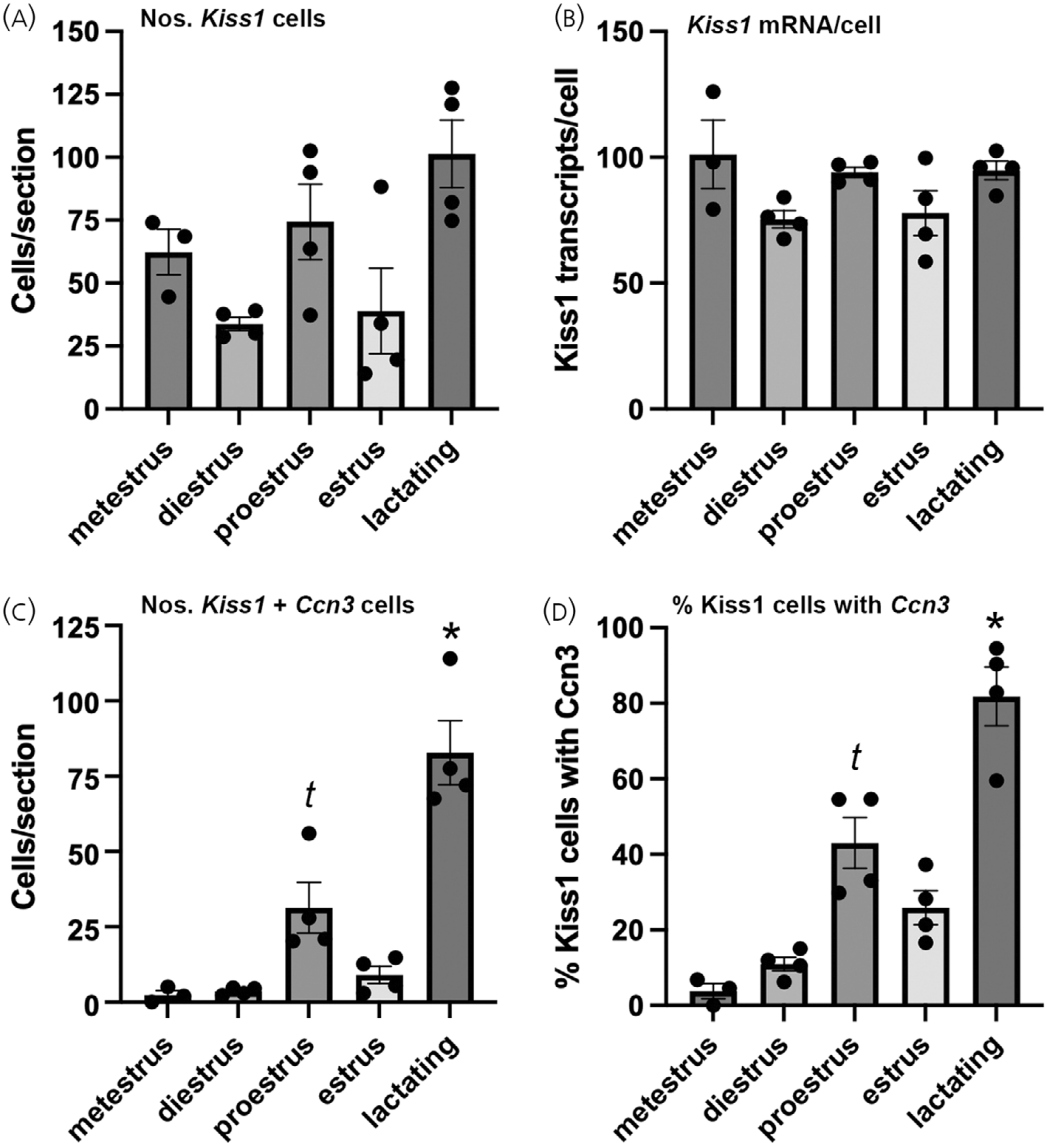
Expression of *Ccn3* and *Kiss1* mRNA in the ARN neurons across the estrous cycle and at lactation (day 11). (A) Mean ± SEM number of cells in ARN with *Kiss1* transcripts. (B) Mean ± SEM number of *Kiss1* RNAscope dots(transcripts)/cell in ARN. (C) Mean ± SEM number of cells expressing both *Kiss1* and *Ccn3* transcripts. *t p* < .05 proestrus versus metestrus and diestrus across all estrous cycle data, *p < .05 lactating versus metestrus and diestrus across all estrous cycle stages. (D) Mean ± SEM percentage of *Kiss1* neurons with *Ccn3* transcripts. *t p* < .05 proestrus versus metestrus and diestrus across all estrous cycle data, *p < .05 lactating versus metestrus and diestrus. In all cases *N* = 4 except for metestrus (*N* = 3). Kruskal–Wallis with post‐hoc Dunn's tests.

Neurons positive for *Kiss1* transcripts were restricted to the ARN (Figure 5). Across all stages of the estrous cycle (*N* = 3‐4/stage), the mean (±SEM) number of detected *Kiss1* mRNA‐expressing cells was 51.7 ± 7.2/section, similar to that of a prior *Kiss1* RNAScope analysis of the ARN in female mice.^23^ Also consistent with previous studies,^24^, ^25^ we found that neither the number of *Kiss1* mRNA‐positive cells nor their transcript levels per cell changed significantly across the estrous cycle or at day 11 of lactation (Figure 6A,B).

The multiplexed RNAscope hybridization revealed that up to one half of all *Ccn3*‐expressing cells in the ARN were *Kiss1* neurons (Figure 5). During lactation, when the highest *Ccn3* signal was observed, 178.2 ± 34.3 Ccn3 positive cells were detected per section and 82.7 ± 10.6 of these were *Kiss1*‐positive neurons. Significant fluctuations in *Ccn3* expression were detected within *Kiss1* neurons across the estrous cycle (Figure 6C,D). When comparing only between cycling mice, a significant increase in the number of dual‐expressing *Ccn3/Kiss1* cells was found on proestrus (*N* = 4) compared to metestrus (*N* = 3) and diestrus (*N* = 4) (Kruskal–Wallis 10.62, *p* = .0017, Dunn's *p* = .0237 (metestrus), *p* = .0482 (diestrus), Figure 6C). The same was observed when analyzing the percentage of *Kiss1* neurons expressing *Ccn3* transcripts (Kruskal–Wallis 12.13, *p* < .0001, Dunn's *p* = .0107 (metestrus), Figure 6D) with ~4% of Kiss1 neurons expressing *Ccn3* transcripts in metestrus compared with 43% in proestrus (Figure 6D).

When comparing across the complete female data set, a robust increase in the numbers of cells expressing both *Ccn3* and *Kiss1* mRNA was found in lactating mice (Kruskal–Wallis 15.88, *p* = .0032, Dunn's *p* = .0051 (metestrus), *p* = .0288 (diestrus), Figure 6C). In metestrus (*N* = 3) and diestrus (*n* = 4), 3.8 ± 1.9% and 10.9 ± 1.8% of Kiss1 neurons expressed *Ccn3* mRNA, respectively, compared with 81.8 ± 7.8% in lactating mice (*N* = 4) (Kruskal–Wallis 16.66, *p* = .0022, Dunn's *p* = .0042 (metestrus), *p* = .0208 (diestrus), Figure 6D).

## 4 DISCUSSION

Previous studies have shown that CCN3 is widely expressed in glia and neurons of the developing forebrain before becoming restricted primarily to the cerebral cortex, hippocampus, and amygdala of adult rodents and humans.^26^, ^27^, ^28^ We observed the same distribution in adult mice but note that low levels of expression persist in other brain regions. Whether CCN3 has a unified role in modulating extracellular signaling in the forebrain or is co‐opted to perform phenotype‐specific functions is unknown. Whereas CCN3 has multiple roles during neural development in processes such as cell adhesion, migration, and survival, its functions in adult neurons and glial cells remain unknown.^27^

We find that the expression of *Ccn3* mRNA in ARN^KISS^ neurons fluctuates across the estrous cycle. Although values for the whole of the ARN failed to reach statistical significance, the numbers of *Ccn3*‐expressing kisspeptin neurons were significantly elevated on proestrus. During metestrus approximately 4% of kisspeptin neurons express *Ccn3* transcripts and this rises to 43% at proestrus. Operating as the GnRH pulse generator, the synchronized activity of the ARN^KISS^ neurons does not change substantially from metestrus to proestrus but then declines markedly at estrous.^29^, ^30^ The role of the proestrous peak in *Ccn3* mRNA is unknown but might possibly result in elevated levels of CCN3 protein on estrous and, conceivably, this may be involved in the slowing of ARN^KISS^ neurons synchronization events and pulsatile LH secretion. However, any role for CCN3 is unlikely to be essential as the embryonic knockout of *Ccn3* is not reported to impair fertility in mice.^31^

A clear sex difference exists in the expression of *Ccn3* mRNA within the mediobasal hypothalamus. Whereas *Ccn3* transcripts are abundant in the cortex, hippocampus, and amygdala of both sexes, *Ccn3* mRNA expression in the mediobasal hypothalamus was only detected in female mice. This sex difference may be reflected in the observation that CCN3 immunoreactivity in the ARN is only detectable in female *Esr1* ^*Nkx2.1‐cre*^ mice, although CCN3 protein expression was not detected in the ARN of either sex in control mice.^10^ There are sex differences in the ability of ESR1‐expressing cells to modulate bone density^11^ and sex differences in hypothalamic CCN3 may play a role in this.^10^

We observed that the ARN, DMN, and MEPD all exhibit a very substantial increase in the expression of *Ccn3* mRNA at day 11 of lactation. This was found to result from an up to 6‐fold increase in the numbers of cells expressing *Ccn3* mRNA without any change in *Ccn3* mRNA levels per cell. The uniformly large increment in the numbers of *Ccn3* mRNA‐expressing neurons in the ARN, DMN, and MEPD suggests a common regulatory mechanism during lactation. However, it is difficult at present to predict what the key regulatory factors may be. There are substantial changes in prolactin and progesterone during lactation^32^ and receptors for estrogen, progesterone, and prolactin are all found in the ARN, DMN, and MEPD of the mouse.^33^, ^34^, ^35^ Nevertheless, the endocrine changes in lactating and in *Esr1* ^*Nkx2.1‐cre*^ mice, the two known conditions of highly up‐regulated CCN3 expression, are not similar. Whereas prolactin is elevated in lactation^32^ it is much less so in *Esr1* ^*Nkx2.1‐cre*^ mice.^11^ Similarly, while ESR1 signaling is absent in some neurons in *Esr1* ^*Nkx2.1‐cre*^ mice, estradiol levels are elevated during lactation^32^ as well as on diestrus prior to proestrus.^18^ It is possible that independent factors are regulating *Ccn3* mRNA expression in ESR1 knockout and lactating mice. Clearly, future experiments need to be undertaken to address the regulatory factors involved in the brain‐wide up‐regulation of *Ccn3* mRNA expression during lactation.

The observation here of a large increase in *Ccn3* mRNA expression in the ARN is consistent with that of Babey and colleagues reporting that CCN3‐immunoreactivity in the ARN peaks around day 14 of lactation.^10^ However, we show here that this lactational up‐regulation in CCN3 is not restricted to the ARN or indeed to the kisspeptin neurons within the ARN. Multiple neuronal populations upregulate *Ccn3* mRNA expression at lactation, and half of all ARN neurons expressing *Ccn3* transcripts in lactating females are not kisspeptin neurons. The identity of the other ARN neurons is not known, but dopamine neurons have been implicated in the *Esr1* ^*Nkx2.1‐cre*^ mouse bone phenotype^10^ and the deletion of ESR1 selectively from ARN proopiomelanocortin (POMC) neurons also results in increased bone strength.^36^ Thus, the rescue of bone density loss in *Esr1* ^*Nkx2.1‐cre*^ mice by knockdown of *Ccn3* in the ARN may result from modulation of one or several different CCN3‐expressing neuronal phenotypes. It is also notable that the bone density of mice with ESR1 knockdown in the adjacent ventromedial nucleus is not different to that of the ARN ESR1 knockdown,^11^ suggesting an even wider locus of estrogen action within the mediobasal hypothalamus (MBH). Future studies will require the deletion of CCN3 in different MBH neuronal phenotypes in adults to establish those that may be involved in modulating bone density.

The mechanism through which ARN neurons may contribute to plasma CCN3 levels requires clarification. It has been proposed that ARN^KISS^ neurons secrete CCN3 into the median eminence to modulate bone density at lactation.^10^ However, studies in rats and mice have shown that the ARN^KISS^ neurons do not project outside the blood‐brain barrier and, as such, do not have direct access to the median eminence.^17^, ^37^ Furthermore, ARN^KISS^ neurons become functionally inactive during lactation, with a near complete absence of their synchronized episodic firing.^38^ It also remains unclear how a small population of kisspeptin neurons could secrete sufficient CCN3 into the median eminence to impact the circulating CCN3 derived primarily from fat stores. For example, even the very substantial secretion of GnRH into the median eminence for over 12 h at the time of the GnRH surge is not itself measurable in the peripheral circulation.^39^ Again, establishing any contribution of ARN^KISS^ neurons to plasma CCN3 concentrations will likely require the selective deletion or knockdown of *Ccn3* in ARN^KISS^ neurons in adult female mice.

In summary, we report here that *Ccn3* is widely expressed in the forebrain of adult mice in a sexually dimorphic manner and that the numbers of cells expressing *Ccn3* transcripts in the ARN, DMN and MEPD are all increased substantially during lactation. We also detect a rise in *Ccn3* mRNA levels in the ARN^KISS^ neurons at the proestrus stage of the cycle. Within the ARN, multiple neural phenotypes express *Ccn3* transcripts during lactation, with approximately one half of these being kisspeptin neurons. The identity of ARN and other mediobasal hypothalamic neurons involved in the estrogen regulation of bone density requires further investigation.

## AUTHOR CONTRIBUTIONS

**Shel‐Hwa Yeo:** Methodology; investigation; formal analysis; data curation. **Zulfiye Gul:** Investigation. **Ziyue Zhou:** Investigation; formal analysis. **Leila Muresan:** Software. **Ellen G. Wall:** Investigation. **Allan E. Herbison:** Conceptualization; writing – review and editing; writing – original draft; supervision; project administration; funding acquisition.

## CONFLICT OF INTEREST STATEMENT

The authors declare no conflicts of interest.

## PEER REVIEW

The peer review history for this article is available at https://www.webofscience.com/api/gateway/wos/peer‐review/10.1111/jne.70050.

## ETHICS STATEMENT

All experimental protocols were approved by the University of Cambridge Animal Welfare and Ethics Review Body under the UK Home Office license P174441DE.

## Data Availability

All primary data will be available from the University of Cambridge Apollo repository (https://www.repository.cam.ac.uk). RNAscope pipeline code is available at https://github.com/lemur01/CountRNAScope.
